# Incidence of pneumonia and risk factors among patients with head and neck cancer undergoing radiotherapy

**DOI:** 10.1186/1471-2407-13-370

**Published:** 2013-08-04

**Authors:** Chin-Nan Chu, Chih-Hsin Muo, Shang-Wen Chen, Shu-Yu Lyu, Donald E Morisky

**Affiliations:** 1 Department of Radiation Oncology, China Medical University Hospital, No. 2, Yuh-Der Road, Taichung 404, Taiwan; 2 Management Office for Health Data, China Medical University Hospital, Taichung 404, Taiwan; 3 College of Medicine, China Medical University, Taichung 404, Taiwan; 4 School of Public Health, Taipei Medical University, 250 Wu-Hsing Street, Taipei 110, Taiwan; 5 Department of Community Health Sciences, Fielding School of Public Health, University of California at Los Angeles, Los Angeles, CA 90095, USA; 6 School of Medicine, Taipei Medical University, 250 Wu-Hsing Streeet, Taipei 110, Taiwan

**Keywords:** Head and neck cancer, Radiotherapy, Pneumonia, Risk factors

## Abstract

**Background:**

This study investigated the incidence and patient- and treatment-related risk factors related to pneumonia acquired during radiotherapy (PNRT) in head and neck cancer (HNC) patients.

**Methods:**

Using the universal insurance claims data, 15,894 total HNC patients between 1998 and 2007 were included in this analysis. PNRT was defined as the occurrence of pneumonia within 90 days of the commencement of radiotherapy. Information also included some demographic characteristics, treatment-related factors, and comorbidities. Appropriate statistical tests were performed to assess the difference between patients with and those without PNRT. A logistic regression was used to estimate the odds ratio (OR) of PNRT among the variables examined.

**Results:**

In total, 772 patients (4.86%) were identified with PNRT as the case group, whereas 15,122 subjects of the same cancer without PNRT formed the control group. Of patients with PNRT, 632 (81.9%) were hospitalized with a mean length of stay of 25.9 days. Results from the multiple logistic regression showed that an older age and certain comorbidities were associated with an increased risk of PNRT. Patients with cancer of the tongue, buccal mucosa, oropharynx, and hypopharynx/larynx were at particularly higher risk (OR = 1.28, 1.28, 1.67, and 1.74, respectively). Compared to radiotherapy alone, concurrent chemoradiotherapy had no effect on the PNRT. Patients in the PNRT group had higher overall medical costs and length of stay.

**Conclusion:**

The incidence of PNRT in HNC patients receiving radiotherapy was approximately 5%. Notably, an older age, certain comorbidities, and certain specific tumor sites were associated with an increased risk.

## Background

It was recently estimated that there are more than half a million new cases of head and neck cancer (HNC) diagnosed annually worldwide [1]. Over the past few decades, radiotherapy (RT) or concurrent chemoradiotherapy (CCRT) has become the standard of care for HNC patients as definitive or adjuvant treatment. Previous studies have reported HNC patients to be at a greater risk of aspiration pneumonia on account of the severe dysfunction of the tongue, larynx, and pharyngeal muscles following RT [2-5], with 33-81% of HNC patients having been reported to suffer aspiration pneumonia during RT therapy [3,5-10].

In addition to being the most common cause of infectious death among cancer patients [11], an episode of pneumonia can severely impact the course of RT by requiring treatment interruption or prolongation, thus jeopardizing local control [12]. While However, to date no population-based estimates of pneumonia incidence during RT have been reported and there is a paucity of data regarding the association between pneumonia and certain factors such as demography, tumor site, and comorbidities.

Therefore, this study set out to estimate the incidence of pneumonia acquired during RT (PNRT) and to explore its risk factors in HNC patients. We utilized data sourced from Taiwan’s universal national health insurance program to perform a population-based analysis investigating the associations between the occurrence of PNRT and various demographic factors and co-morbidities. These results will help establish a risk model and prioritize preventive efforts for those at greatest risk.

## Methods

### Data source

The Taiwan Department of Health combined 13 insurance systems into a universal National Health Insurance program in March 1995. Approximately 99% of the population has been covered by this system since 1999. The National Health Research Institute (NHRI) produces computerized medical claims and selected sets of healthcare data for administrative use and research, as described in a large retrospective study [13,14]. We used the NHRI data from 1998 to 2007, including all inpatient and ambulatory care records for cancer care and a registry of catastrophic illness patients, which also contain basic demographic information of insured residents. We used the International Classification of Disease, 9th Revision, Clinical Modification (ICD-9-CM) to identify the disease and retrieve information on diagnoses of these patients. Because the NHRI database provided by the official NHI program consists of totally de-identified, encrypted, secondary data released to the public for research purposes without personal or institutional identification or contact with the study patients, the study was exempt from full review by the institutional review board (IRB) of China Medical University Hospital.

### Study subjects

We selected 27,617 newly diagnosed patients with HNCs, (ICD-9-CM 140~149) from the registry for catastrophic illness patients between 1998 and 2007. All patients were treated with RT (ICD-9-CM-OP 922 and V580) after the diagnosis. Locations of the HNCs included the lips (ICD-9-CM 140), tongue (ICD-9-CM 141), salivary glands (ICD-9-CM 142), gums (ICD-9-CM 143), mouth floor (ICD-9-CM 144), buccal mucosa (ICD-9-CM 145), oropharynx (ICD-9-CM 146), nasopharynx (ICD-9-CM 147), hypopharynx (ICD-9-CM 148), and unknown primary (ICD-9-CM 149).

**Figure 1:**
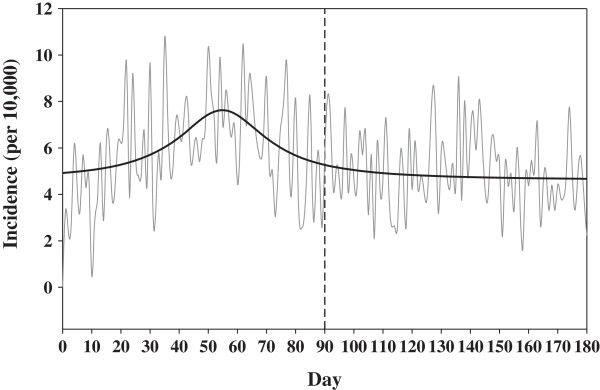
Incidence* of pneumonia in head and neck cancer patients. This figure presents the incidence of pneumonia beginning with the initiation of radiotherapy. The thick line illustrates the incidence.

The first date of RT was defined as the index date. After excluding patients with pneumonia (ICD-9-CM 481~482 and 485~486) before the index date, those younger than 20 years, and patients receiving chemotherapy before RT, 15,894 HNC patients were included in this analysis. As depicted in Figure 1, HNC patients were particularly at risk of pneumonia within 90 days from the index date. Thereafter, the rate of the pneumonia-free proportion stabilized. Thus, the study group was defined as those with the occurrence of PNRT within 90 days after the index date.

Patient information retrieval also included selected sociodemographic characteristics, treatment-related factors, and comorbidities, including age (20–49, 50–64, and ≥ 65 years), gender, level of hospital (medical center, regional hospital, or district hospital), neck lymph node dissection (ICD-9-CM-OP 403~405), a gastrostomy (ICD-9-CM-OP 430, 431, 463, 463.2, and 463.9), chemotherapy (ICD-9-CM-OP V581 and 992.5), dementia (ICD-9-CM 290 and 294), stroke (ICD-9-CM 430~438), gastroesophageal reflux disease (GERD, ICD-9-CM 530.11, 530.81, and 787.1), and Parkinson’s disease (PD; ICD-9-CM 332). All comorbidities were identified before the diagnosis of HNCs. CCRT patients were recognized when chemotherapy was administrated during the RT course. Gastrostomy was adjusted for if performed prior to the index date.

### Statistical analysis

We used SAS software version 9.1 (SAS Institute, Cary, NC) for the analysis. Chi-squared and Fisher’s exact tests were performed to test for differences in sociodemographic characteristics and medical records between HNC patients with and without pneumonia. A *t*-test was utilized to examine differences in age and medical costs between groups. A logistic regression was used to estimate the odds risk (OR) of having PNRT among associated factors, after controlling for certain variables. A two-sided *p* of < 0.05 was considered statistically significant.

## Results

Among 15,894 HNC patients, 772 patients (4.86%) were identified as having PNRT and were labeled the case group, with 15,122 HNC patients without PNRT as the control group. Table 1 summarizes the characteristics of the two groups. Compared to the control group, there were some propensities in the case group, such as older age (median age; 58.0 vs. 51.2 years), treated at medical centers, received pretreatment gastrostomy, and associations with certain comorbidities (including stroke, GERD, and PD). Nevertheless, a lower proportion of subjects in the study group were treated with neck lymph node dissection before RT. As shown in Table 2, the logistic regression showed that an older age, treatment center, a gastrostomy, and comorbidities were associated with an increased risk of PNRT, whereas those receiving neck lymph node dissections had a lower risk. Patients with cancer of the tongue (Odds ratio (OR)=1.28; 95% confidence interval (CI)=1.02~1.61), buccal mucosa (OR=1.28, 95% CI=1.07~1.53), oropharynx (OR=1.67, 95% CI=1.36~2.10), and hypopharynx (OR=1.74, 95% CI=1.37~2.21) were at greater risk of PNRT after adjusting for age, gender, level of hospital, treatment, and comorbidities. In particular, nasopharyngeal cancer patients had a substantially lower risk of PNRT.

**Table 1:** Socio-demographic data between head and neck cancer patients with and without pneumonia acquired during radiotherapy (PNRT)

	**PNRT**				
	**No *N*=15,122**		**Yes *N*=772**		
**Variable**	***n***	**%**	***n***	**%**	***p*value**
Men	12,629	83.5	689	89.3	< 0.0001
Age (years)					< 0.0001
< 50	7635	50.5	238	30.8	
50~64	5320	35.2	287	37.2	
≥ 65	2167	14.3	247	32.0	
Mean (SD)	51.2	(12.1)	58.0	(13.2)	< 0.0001
Hospital level					0.001
Medical center	9799	64.8	523	67.8	
Regional hospital	3475	23.0	189	24.5	
District hospital / clinic	1848	12.2	60	7.8	
Treatment					
Neck lymph node dissection	3066	20.3	123	15.9	0.003
Gastrostomy	182	1.2	28	3.6	< 0.0001
Concurrent chemotherapy	6881	45.5	354	45.9	0.85
Comorbidity					
Dementia	41	0.3	4	0.5	0.28^†^
Stroke	418	2.8	46	6.0	<0.0001
Parkinson’s disease	19	0.1	5	0.7	0.005^†^
GERD	664	4.4	51	6.6	0.004

^†^ Fisher’s exact test.

*GERD* gastroesophageal reflux disease.

**Table 2:** Univariate and multivariate logistic regression estimating odds ratio (OR) of developing pneumonia acquired during radiotherapy (PNRT) among variables

**Variable**	**Univariate OR**	**(95% CI)**	**Multivariate OR**	**(95% CI)**
Male (vs. Female)	1.64	(1.30~2.07)	1.77	(1.40~2.24)
Age (vs. < 50 years)				
50~64	1.73	(1.45~2.06)	1.67	(1.40~2.00)
≥ 65	3.66	(3.04~4.40)	3.54	(2.93~4.28)
Hospital level (vs. District hospital/clinic)				
Medical center	1.64	(1.25~2.16)	1.69	(1.29~2.23)
Regional hospital	1.68	(1.25~2.25)	1.68	(1.25~2.26)
Treatment (vs. no)				
Neck dissection	0.75	(0.61~0.91)	0.77	(0.63~0.94)
Gastrostomy	3.09	(2.06~4.63)	2.94	(1.94~4.44)
Comorbidity (vs. no)				
Stroke	2.23	(1.63~3.05)	1.35	(0.98~1.87)
Parkinson’s disease	5.18	(1.93~13.9)	3.01	(1.09~8.30)
GERD	1.54	(1.15~2.07)	1.48	(1.09~1.99)
Cancer site				
Lip	0.63	(0.23–1.71)	0.63	(0.23–1.73)
Tongue	1.13	(0.91–1.40)	1.28	(1.02–1.61)
Salivary gland	0.74	(0.30–1.81)	0.57	(0.23–1.40)
Gum	0.85	(0.53–1.36)	0.70	(0.43–1.14)
Mouth floor	1.67	(0.94–2.95)	1.75	(0.98–3.13)
Buccal mucosa	1.31	(1.11–1.55)	1.28	(1.07–1.53)
Oropharynx	1.90	(1.54–2.36)	1.67	(1.36–2.10)
Nasopharynx	0.46	(0.39–0.53)	0.46	(0.39–0.54)
Hypopharynx	2.33	(1.86–2.93)	1.74	(1.37–2.21)
Unknown	1.21	(0.65–2.23)	1.10	(0.59–2.04)

*GERD* gastroesophageal reflux disease.

Table 3 summarizes the ORs of PNRT among different treatment combinations compared to RT alone. CCRT was not associated with an increased risk of PNRT (OR = 1.12, 95% CI 0.95~1.32). When neck lymph node dissection was performed before RT, there was a trend toward a lower risk (OR = 0.75, 95% CI 0.58~0.98). In contrast, neck lymph node dissection followed by CCRT did not significantly decrease the risk of PNRT. To determine the cause of this disparity, further analysis investigated sociodemographic differences between those with and without lymph node dissection. The results showed that there was a lower proportion of lymph node dissection in the older age group compared to the younger age group (16.5% vs. 9.8% in patients aged ≥ 65 years, *p* < 0.0001).

**Table 3:** Adjusted odds ratio (OR) of having pneumonia acquired during radiotherapy (PNRT) among different treatment modalities

**Treatment**	***n***	**Cases**	OR*	**(95% CI)**
RT alone	6397	336	1.00	(reference)
Neck lymph node dissection plus RT	1915	72	0.75	(0.58~0.98)
CCRT alone	5961	303	1.12	(0.95~1.32)
Neck lymph node dissection plus CCRT	1274	51	0.88	(0.65~1.20)

*: After controlling for gender, age, level of hospital, stroke, gastroesophageal reflux disease, and Parkinson’s disease.

*CCRT* concurrent chemoradiotherapy, *RT* radiotherapy.

A cost analysis was carried out to estimate the impact of PNRT on hospital resources spent, and results are shown in Table 4. Based on calculations of the average medical expenses within 90 days after the index date, there was a significant difference between patients with and without PNRT. Of patients with PNRT, 632 (81.9%) were hospitalized with a mean length of stay of 25.9 days. Those with PNRT spent an additional US$11,612 (approximately US$188/day) on overall medical costs (*p* < 0.0001) and had an extra 5 days for the LOS (*p* < 0.0001).

**Table 4:** Medical costs* between groups with and without pneumonia acquired during RT (PNRT) by t-test

	**PNRT**						
	**No**			**Yes**			
**Variable**	***N***	**Mean**	**SD**	***N***	**Mean**	**SD**	***p*value**
Overall medical costs (US$)	15,122	9744.7	4729.5	772	11,612.3	7520.5	< 0.0001
Average daily medical costs (US$)	15,122	119.8	66.1	772	187.9	98.3	< 0.0001
Length of stay	8257	22.9	29.6	693	27.6	26.0	< 0.0001

*: Cost calculated for each patient within 90 days from the start of radiotherapy.

## Discussion

In HNC patients, more-aggressive treatment might produce unintended consequences such as an increased risk for pneumonia. To the best of our knowledge, this study is the first population-based analysis incorporating demographic, treatment-related factors, tumor sites, and comorbidities to investigate the incidence of PNRT and its risk factors. Our results demonstrate that HNC patients are at particularly high risk of pneumonia within three months following the initiation of RT.

These findings highlight a challenge for radiation oncologists as pneumonia has a negative effect on RT, and often necessitates treatment interruption or prolongation, thus jeopardizing local control and raising the risk of tumor repopulation [12]. Therefore it is the hope of the authors that these results be used to help optimize treatment outcomes.

Patients with some specific tumor sites were at a particularly increased risk for pneumonia, which offers further support for some underlying mechanisms and may indicate the how clinical improvements may be directed. This information is also useful to clinicians who may consider increased surveillance on higher risk individuals.

After stratifying by cancer site, our data indicated that patients with hypopharyngeal, oropharyngeal, tongue, and buccal cancers were at a 1.28~1.74-fold higher risk of developing PNRT. This is in line with previous studies which have suggested HNC patients to be at a greater risk of aspiration pneumonia on account of the severe dysfunction of the tongue, larynx, and pharyngeal muscles following RT [2-5,15]. For pharyngeal cancers, the close proximity of the pharyngeal constrictors to the tumor causes patients to be susceptible to dysphagia, with dysphagia having been demonstrated to carry an increased risk of aspiration [16].

These results also support those of our previous study [17] in which we found maximal dysphagia score during CCRT to be a prognostic factor in predicting early termination of treatment. Thus, careful monitoring of aspiration pneumonia is essential for patients in whom dysphagia worsens during the RT/CCRT course.

We further feel that these data support the use of novel RT techniques to spare the swallowing structures in terms of avoiding aspiration pneumonia during RT and preventing long-term complications [3,5,6,18,19]. Two studies have already been conducting indicating that by sparing the pharyngeal constrictors the incidence of severe dysphagia can be reduced during the RT course [8,18].

The present study was also the first to identify patients with tongue or buccal cancers to be at an increased risk of developing PNRT. Because most patients with these cancers are treated with wide excision and reconstruction, one reason might be attributed to the fact that many reconstruction procedures cannot entirely compensate for swallowing dysfunction. As a result, some patients are still susceptible to both dysphagia and aspiration during the RT course.

Our results showed that patients pretreated with a gastrostomy had an increased risk of PNRT, whereas RT alone plus neck lymph node dissection lowered the hazard. The former was probably due to a selection bias, because those experiencing severe dysphagia are always advised to have a feeding gastrostomy. The latter might be attributed to a predisposition for an uneven age distribution between patients with or without neck node dissection.

As several studies have pointed out that pneumonia has become another major source of post-CCRT morbidity in HNC patients [5,8,15,20-22], we further analyzed our data to estimate the association between pneumonia and post-CCRT. However, in contrast with one study performed by Francis et al. utilizing SEER data to analyze dysphagia, stricture, and pneumonia among HNC patients [15], our study failed to detect an increased incidence of PNRT among CCRT patients when compared with patients undergoing RT alone, despite the increased toxicity accompanying CCRT.

There are two plausible explanations for this disparity. First, patients vulnerable to pneumonia during CCRT may be more likely to receive more comprehensive medical care. For example, drug-related vomiting has been greatly reduced through using novel antiemetics which very well may also reduce the risk of aspiration. Second, previous studies indicated that among patients receiving CCRT, the incidence of post-treatment dysphagia and stricture was considerably higher as a long-term or late complication rather than as an early event. As the Francis et al. study mentioned above tracked changes in rates over seven years, and this study only had a three month follow-up time, the Francis et al. study was far better positioned to detect this sort of late complication.

With a trend toward expanding RT/CCRT for HNCs, it is important to initiate a preventive strategy for all involved practitioners when counselling patients before RT. Particularly, a cost-investigation study showed that morbidity and length of stay (LOS) could have significant impact on hospital resources spent on in-patients with cancer [23]. Since cancer patients may have higher medication costs, it is imperative to determine who is at risk of developing morbidities during treatment.

This retrospective study should be interpreted in light of several limitations. First, the data analyzed in this study was gathered from an administrative database and lacks some important clinical and lifestyle information. For example, we were unable to adjust for smoking which likely had an effect on both the development of cancer and pneumonia. Also, we were unable to incorporate stage data into this analysis. Furthermore, as the NHRI data do not provide comprehensive information regarding the pathogen causing the pneumonia, there was no clear delineation of the risks between cases of PNRT from aspiration and those due to an immunocompromised status. Although a diagnosis of pneumonia is objective and is usually attributable to aspiration in patients treated for HNC, some might also be vulnerable to pneumonia due to an immunocompromised status following aggressive therapy [11]. Based on clinical manifestations or radiological patterns, it is not possible to entirely distinguish differences between the two causes of pneumonia.

However, we feel that the vast majority of the PNRT subjects in this study were aspiration cases. According to our previous experience [17], the incidence of aspiration pneumonia during a CCRT course exceeds that of acquired pneumonia due to the later circumstance by about 4-fold. Furthermore, patients with an extreme neutropenic status, such as those undergoing chemotherapy, are likely to have poor outcomes such as increased infections. However, given the poor immune status of these individuals, the body is unable to effectively mount a strong immune response despite the presence of a foreign pathogen. This phenomenon would work deter the clinician’s attempts at detecting the infection and lead to an underestimation of aspiration. Nonetheless, both events can lead to catastrophic effects on treatment outcomes.

Dysphagia is a common consequence during an RT course. No specific coding for dysphagia was identified in the NHRI data; therefore, it was not possible to correlate dysphagia with pneumonia in this study. Also, the impact of dysphagia/dysmotility, secondary to excision of primary tumors, could not be assessed because of the diversities of coding numbers of the NHRI data. Thus, further validation will be required by examining information pertaining to postoperative swallowing function.

## Conclusions

HNC patients were at particular risk of pneumonia within 90 days from the start of RT, and the estimated incidence was approximately 5% in this period. Irrespective of the cause of the pneumonia, old age, comorbidities, and certain tumor sites were associated with an increased risk. Special preventive efforts should be taken during the RT course for those at greater risk.

## Competing interests

The authors declare that they have no competing interests.

## Authors’ contribution

C-NC, Designed the study and drafted the manuscript. V-HM, Performed the data analysis and drafted the manuscript. S-WC, Supervised the research team. Critically reviewed the manuscript. S-YL, Assisted in drafting the manuscript, and critically reviewed the manuscript. DEM, Critically reviewed the manuscript. All the authors have read and approved the final version of this manuscript.

## Pre-publication history

The pre-publication history for this paper can be accessed here:

http://www.biomedcentral.com/1471-2407/13/370/prepub
